# Chasing genetic correlation breakers to stimulate population resilience to climate change

**DOI:** 10.1038/s41598-022-12320-3

**Published:** 2022-05-17

**Authors:** Jaroslav Klápště, Emily J Telfer, Heidi S Dungey, Natalie J Graham

**Affiliations:** Scion New Zealand Forest Research Institute Ltd., Rotorua, 3046 New Zealand

**Keywords:** Heritable quantitative trait, Plant breeding, Population genetics

## Abstract

Global climate change introduces new combinations of environmental conditions, which is expected to increase stress on plants. This could affect many traits in multiple ways that are as yet unknown but will likely require the modification of existing genetic relationships among functional traits potentially involved in local adaptation. Theoretical evolutionary studies have determined that it is an advantage to have an excess of recombination events under heterogeneous environmental conditions. Our study, conducted on a population of radiata pine (*Pinus radiata* D. Don), was able to identify individuals that show high genetic recombination at genomic regions, which potentially include pleiotropic or collocating QTLs responsible for the studied traits, reaching a prediction accuracy of 0.80 in random cross-validation and 0.72 when whole family was removed from the training population and predicted. To identify these highly recombined individuals, a training population was constructed from correlation breakers, created through tandem selection of parents in the previous generation and their consequent mating. Although the correlation breakers showed lower observed heterogeneity possibly due to direct selection in both studied traits, the genomic regions with statistically significant differences in the linkage disequilibrium pattern showed higher level of heretozygosity, which has the effect of decomposing unfavourable genetic correlation. We propose undertaking selection of correlation breakers under current environmental conditions and using genomic predictions to increase the frequency of these ’recombined’ individuals in future plantations, ensuring the resilience of planted forests to changing climates. The increased frequency of such individuals will decrease the strength of the population-level genetic correlations among traits, increasing the opportunity for new trait combinations to be developed in the future.

## Introduction

Global climate change will likely introduce new combinations of environmental conditions into our forest systems, increasing the physiological stress affecting plants and affecting multiple traits in multiple ways. Coping with these ecological changes in the long-term may require the modification of the underlying genetic correlation matrix among functional traits^1^. Genetic correlations are pairwise trait associations based on pleiotropic mutations^2^, and linkage disequilibrium (LD) (co-segregation of quantitative trait loci (QTLs))^3^. Pleiotropic mutations can be present at the allelic level (single causal variant contributes to multiple phenotypes) or at the gene level (multiple causal variants within a gene contributing to multiple phenotypes)^2^ and appear to be the primary cause of the genetic correlations observed among traits in populations under random mating^4,5^. Linkage disequilibrium or a shared environment can also contribute to genetic correlations in a population under non-random mating with the presence of inbreeding^6^.

Genetic correlations represent evolutionary constraints that have developed over time, and their values and directions can vary according to differences in micro-evolutionary processes along environmental gradients^1,7^. Alternatively, high positive genetic correlations between functional traits provide a mean for their co-evolution^8^. Sgró and Hoffmann^9^ found changes in environmental conditions to be the cause of changes in the value and direction of genetic correlations in life history traits that were observed in samples from the same population, stressing the need to investigate genetic correlations among different sets of environmental conditions. Previous studies found substantial changes in genetic parameters when estimated in favourable versus stressful environmental conditions^10,11^. Although the presence of high recombination through migration might be counteracting local adaptation to the current environmental conditions^12^, theoretical evolutionary genetics studies have found the evolution of high recombination rates when selection targeted different loci under heterogeneous environmental conditions^13,14^. Thus, high recombination rates are crucial in adaptation under rapidly changing environments.

Adverse genetic correlations constraining genetic improvement progress can similarly be found in breeding populations^15^. In these circumstances, the undesirable genetic patterns between traits can be challenged by specific breeding (complementary mating) and/or selection procedures in order to select ’correlation breakers’ (individuals from the studied population that strongly deviate from the general trend between investigated traits) and eliminate the unfavourable trends^15^. One of the most critical adverse genetic correlation in forest trees is the relationship between tree productivity and wood density. This correlation is often strongly negative e.g. $\sim$ − 0.51^16^. Additionally, both traits seem to be involved in adaptive processes. While productivity in terms of growth speed is considered a means to deal with competition for the sun light from surrounding plants^17^, wood density is related to resistance to drought stress, through thicker cell walls which prevent cell implosion due to higher negative pressure in the xylem pipelines or embolism^18,19^, and to wood stiffness^20^. Breaking this strongly negative genetic correlation could unlock the genetic potential of trees. Therefore, finding individuals with a much greater recombination rate than normal is strategy to explore the potential of this approach.

Unravelling the mechanisms underlying the observed phenotypic correlations among traits is one of the greatest challenges in quantitative genetics^15^. A phenotypic correlation is the product of genetic and environmental correlations and their interplay^21^. Genetic correlations represent associations among the genetic causes of the traits investigated, which can be affected by many factors such as sampling error, environmental heterogeneity and developmental stage. Genetic correlations can also vary with the expression of different genes in different environments or developmental phases^22^. While genetic correlations reflect general trends in populations, they can also be modified by the selection of correlation breakers to shift the association between traits in a favourable direction^15^.

Current progress in next-generation sequencing has enabled the development of genomic resources for non-model organisms and allowed the closer investigation of correlations among genotypes and phenotypes through association mapping^23–25^. Such analyses can be used effectively to select recombinants of known quantitative trait nucleotides (QTNs) to break adverse genetic correlations, especially in cases where genetic correlations are built-up through chromosomal linkages rather than pleiotropic effects^26^.

Genetic analyses investigating single-trait versus multi-trait models have found that multi-trait models were superior in the accuracy of estimated genetic parameters such as additive genetic variance, heritability and breeding values, particularly where there are significant genetic correlations between traits^27,28^. However, multi-trait prediction models appear to be less advantageous when traits are uncorrelated, even proving inferior to single-trait models. Additionally, the multi-trait models are not useful for identifying individuals that break unfavourable genetic correlations^27^.

Our analysis investigates the genomic features of genetic correlation breakers and the ability to predict these types of individuals using genomics. We propose that genomics will allow us to select individuals with high level of recombination and that these individuals will have favourable combinations of traits that are otherwise negatively correlated at the population level. We used a radiata pine (*Pinus radiata* D. Don) population established using tandem selection based on New Zealand’s nation-wide breeding values as a test case^15^. Using genomic data, we identified individuals that were correlation breakers for growth and wood density traits, and used genomic data to test for high levels of recombination in these individuals. We also investigated how recombination within the population described differs with a another reference population of the same species. With the knowledge that recombination and heterogeneity has been associated with genetic robustness^14^, we discuss the implications of our results on the potential to select for genotypes that will remain robust under future climate change.

## Materials and methods

### Plant material

The New Zealand (NZ) Radiata Pine Breeding Company’s (RPBC) breeding population includes populations selected for either high wood density or growth and form attributes, using NZ nation-wide breeding values that reflect a common genetic effect across tested environments. The breeding program strategy proposed further developments are outlined in detail in previous studies^29,30^. Briefly, the program is based on an open nucleus breeding strategy with two independent sublines. The main population within each sublines is structured into different breeding goals such as growth and form, long internode, high wood density, structural timber, and tested as an open pollinated population. The elite population comprises genetically narrow material that is tested through control pollinated progenies, with or without vegetative propagation (clonal trials). Selection is usually performed on the basis of a ’growth and form’ (GF) score, which is an artificially created scale from 7 (unimproved) to 30 (highly improved) representing weighted breeding values for growth and form attributes^31^.

The sample under study was composed of two populations: POP1 was used as the training population, and POP2GF was the population in which to predict individuals with excess recombination using a genomic prediction model. All trials were clonal full-sib families and replicated among sites at Tarawera, Woodhill and Kinleith in the North Island of New Zealand. The POP1 population was established from parents chosen through two selection strategies. One was based on a tandem selection, where 19 parents were first selected for growth and form using GF scores followed by a second round of selection focused on high wood density (HD). This group (POP1HD) formed a population of 160 individuals termed ’correlation breakers’. The second selection strategy in POP1 was based purely on GF scores where 33 parents were selected to create POP1GF, consisting of 304 individuals. POP1GF and POP1HD were established using a single-pair mating design which produced 33 (POP1GF) and 19 (POP1HD) full-sib families. Both populations were planted at Tarawera using a single tree plot, set within replications design with six replications. Each family was represented by 10 genotypes and each genotype was tested in six copies.

The POP2GF population, consisting of 523 individuals, was also selected based only on GF scores. POP2GF was established using a factorial mating design which produced 42 full-sib families from 24 parents. The POP2GF population was planted across two sites, Woodhill and Kinleith, using an incomplete block design with five replications and nine incomplete blocks (each representing six families) within each replication. Each family included 10 genotypes which were tested in five copies. There were two common parents between POP1GF and POP1HD, 12 common parents between POP1GF and POP2GF and one parent between POP1HD and POP2GF. Full trial details are reported in Li et al.^32^.

All 987 clonally replicated genotypes were measured for the traits branch cluster (BR9), using a 9 points scale from 1 (uninodal) to 9 (extreme multinodal)^33^, straightness (ST9), using a 9 points scale from 1 (crooked) to 9 (very straight)^34^, diameter at breast height (DBH [cm]), and wood density (WD [${\text{kg/m}}^{3}$]) measured as basic wood density through the maximum moisture content method^35^.

### Genomic resources

Genomic data were generated using a previously developed exome capture—genotyping by sequencing platform^36^ as described in Telfer et al.^37^. In brief, transcriptomic resources, that represented gene expression across broad range of tissues, including compression wood xylem, spring xylem, summer xylem, summer phloem, spring buds, autumn buds, healthy needles, needles infected by *Phytophtora pluvialis*, seedling phloem and seedling xylem^37^, were aligned to *Pinus taeda* reference genome v. 1.01e and used to develop 120 base capture probes. Captured markers were removed if heterozygosity shown in megagametophyte tissues was higher than 5%, average read depth was less than 10, multiple alleles were detected, or only singletons were observed. Additionally, individual datapoints were classified as missing if the ratio between reference and alternative allele was lower than 0.1 and the number of read was less than 10^38^. The average read depth was 59.2 per marker and 59.04 per individual. Data were further filtered for minor allele frequency (MAF) > 0.01 and missing data were imputed with mean genotype. Total number of markers was 80,159 SNPs after filtering.

### Genomic data analysis

The analysis of linkage disequilibrium (LD) was based on composite LD correlation ’r’ equivalent to the Pearson’s product-moment correlation coefficient between genotypes of investigated loci^39^. To reduce bias in estimation of LD due to familial structure, we implemented LD corrected for relatedness as proposed by Mangin et al.^40^ as follows:

$$\begin{aligned} {\hat{r}}_v(i,j)=\frac{\left( \sum ^G_{X^i,X^j}\right) ^2}{\sum ^G_{X^i,X^i}\sum ^G_{X^j,X^j}} \end{aligned}$$

where $\sum _{X^i,X^j}^G$ is the sample variance-covariance matrix defined as $([{\varvec{X}}^i,{\varvec{X}}^j ]-\frac{(1_N 1_N^T {\varvec{G}}^{-1})}{(1_N^T {\varvec{G}}^{-1} 1_N )} [{\varvec{X}}^i,{\varvec{X}}^j ]) {\varvec{G}}^{-1} ([{\varvec{X}}^i,{\varvec{X}}^j ]-\frac{(1_N 1_N^T {\varvec{G}}^{-1})}{(1_N^T {\varvec{G}}^{-1} 1_N )} [{\varvec{X}}^i,{\varvec{X}}^j ])$, where ${\varvec{X}}^i$ and ${\varvec{X}}^j$ are the vectors of genotypes for $i^{th}$ and $j^{th}$ marker, N is the sample size and ${\varvec{G}}$ is the marker-based relationship matrix^41^. The analysis was performed using ’LDcorSV’ R package^42^. Additionally, the genomic differences between POP1HD (correlation breakers) and POP1GF populations were investigated through comparison of the mean observed and expected heterozygosity (approximating effective population size) using *t* test^43^. The 100 individuals randomly selected from each population were used to estimate the sample mean observed heterozygosity, which was performed 100 times. The trend line of decay in LD was estimated using the Hill and Weir expectation^44^ as follows: $E(r^2 )=[\frac{10+C}{(2+C)(11+C)]}[1+\frac{(3+C)(12+12C+C^2)}{n(2+C)(11+C)}]$ where n is sample size and C is the parameter to be estimated and represents the product of the population recombination parameter (C $=$ 4Nc), where N is the effective population size and c the recombination rate. The nonlinear least squares were used to fit the data using ’nls’ R package^45^. The statistical significance in LD pattern difference between POP1GF and POP1HD in the training population was investigated through a Jennrich test^46^, testing the null hypothesis that the two correlation matrices are not different from each other. Only scaffolds with at least three overlapping markers between samples were included in this analysis.

### Statistical analysis

Phenotypic data were standardised for each trait at each site to avoid the problems associated with combining breeding values with different scales into a multi-trait selection index. The ’ASReml-R’ package^47^ was used to estimate genetic parameters such as variance components, heritability, and genetic correlations. The multivariate linear mixed model was implemented in the POP1 populations (POP1HD and POP1GF) as follows:

$$\begin{aligned} {\varvec{Y}}={\varvec{X\beta }}+{\varvec{Za}}+{\varvec{Zg}}+{\varvec{Zr}}+{\varvec{Zs}}+{\varvec{e}} \end{aligned}$$

where ${\varvec{Y}}$ is the matrix of measurements, ${\varvec{\beta }}$ is the vector of fixed effects (intercept), ${\varvec{a}}$ is the vector of genomic estimated breeding values following var(${\varvec{a}}$)$\sim$N(0,G1) where G1 is the variance-covariance structure for genomic estimated breeding values following

$$\begin{aligned} G1=\begin{bmatrix} \sigma _{a_1}^2 &{}\ldots &{}\sigma _{a_1 a_n} \\ \vdots &{}\ddots &{}\vdots \\ \sigma _{a_n a_1} &{}\ldots &{}\sigma _{a_n}^2\\ \end{bmatrix}\otimes {\varvec{A}} \end{aligned}$$

where ${\varvec{A}}$ is the average numerator relationship matrix^48^, $\sigma _{a_1}^2$ and $\sigma _{a_n}^2$ are additive genetic variances for the 1st and *n*th trait, $\sigma _{a_1 a_n}$ and $\sigma _{a_n a_1}$ are additive genetic covariances between the 1st and *n*th trait, and $\otimes$ is the Kronecker product. The marker-based analysis was performed by substituting the pedigree-based relationship matrix ${\varvec{A}}$ with the marker-based relationship matrix ${\varvec{G}}$, estimated following^41^:

$$\begin{aligned} {\varvec{G}}=\frac{{\varvec{ZZ'}}}{2\sum _ip_i (1-p_i)} \end{aligned}$$

where ${\varvec{Z}}$ = ${\varvec{M}} - {\varvec{P}}$, where ${\varvec{M}}$ is a matrix of genotypes coded 0, 1 and 2, indicating the number of alternative alleles in the genotype (relative to the loblolly pine (*Pinus taeda*) reference genome v. 1.01e^49^) , ${\varvec{P}}$ is twice the alternative allele, ${\varvec{g}}$ is the vector of random non-additive genetic effects following var(${\varvec{g}}$)$\sim$N(0,G2), where G2 is the variance-covariance structure for non-additive genetic effects following

$$\begin{aligned} G2=\begin{bmatrix} \sigma _{g_1}^2 &{}\ldots &{}\sigma _{g_1 g_n} \\ \vdots &{}\ddots &{}\vdots \\ \sigma _{g_n g_1} &{}\ldots &{}\sigma _{g_n}^2\\ \end{bmatrix}\otimes {\varvec{I}} \end{aligned}$$

where $\sigma _{g_1}^2$ and $\sigma _{g_n}^2$ are non-additive genetic variances for the 1st and the *n*th trait, $\sigma _{g_1 g_n}$ and $\sigma _{g_n g_1}$ are non-additive genetic covariances among the 1st and *n*th trait, ${\varvec{I}}$ is the identity matrix, ${\varvec{r}}$ is the vector of random replication effects following var(${\varvec{r}}$)$\sim$N(0,G3), where G3 is the variance-covariance structure for replication effects following

$$\begin{aligned} G3=\begin{bmatrix} \sigma _{r_1}^2 &{}\ldots &{}\sigma _{r_1 r_n} \\ \vdots &{}\ddots &{}\vdots \\ \sigma _{r_n r_1} &{}\ldots &{}\sigma _{r_n}^2\\ \end{bmatrix}\otimes {\varvec{I}} \end{aligned}$$

where $\sigma _{r_1}^2$ and $\sigma _{r_n}^2$ are replication variances for the 1st and the *n*th trait, $\sigma _{r_1 r_n}$ and $\sigma _{r_n r_1}$ are replication covariances between the 1st and the *n*th trait, ${\varvec{s}}$ is the vector of random set effects nested within replicate effects following var(${\varvec{s}}$)$\sim$N(0,G4), where G4 is the variance-covariance structure for set nested within replication effects following

$$\begin{aligned} G4=\begin{bmatrix} \sigma _{s_1}^2 &{}\ldots &{}\sigma _{s_1 s_n} \\ \vdots &{}\ddots &{}\vdots \\ \sigma _{s_n s_1} &{}\ldots &{}\sigma _{s_n}^2\\ \end{bmatrix}\otimes {\varvec{I}} \end{aligned}$$

where $\sigma _{s_1}^2$ and $\sigma _{s_n}^2$ are set nested within replication variances for the 1st and the *n*th trait, $\sigma _{s_1 s_n}$ and $\sigma _{s_n s_1}$ are set nested within replication covariances between the 1st and the *n*th trait, ${\varvec{e}}$ is the vector of random residuals following var(${\varvec{e}}$)$\sim$N(0,R), where R is the variance-covariance structure for residual effects following

$$\begin{aligned} R=\begin{bmatrix} \sigma _{e_1}^2 &{}\ldots &{}\sigma _{e_1 e_n} \\ \vdots &{}\ddots &{}\vdots \\ \sigma _{e_n e_1} &{}\ldots &{}\sigma _{e_n}^2\\ \end{bmatrix}\otimes {\varvec{I}} \end{aligned}$$

where $\sigma _{e_1}^2$ and $\sigma _{e_n}^2$ are residual variances for the 1st and the *n*th trait, $\sigma _{e_1 e_n}$ and $\sigma _{e_n e_1}$ are residual covariances between the 1st and the *n*th trait, and ${\varvec{X}}$ and ${\varvec{Z}}$ are incidence matrices assigning fixed and random effects to measurements.

The multivariate linear mixed model was implemented in the POP2GF population as follows:

$$\begin{aligned} {\varvec{Y}}={\varvec{X\beta }}+{\varvec{Za}}+{\varvec{Zg}}+{\varvec{Zr}}+{\varvec{Zb}}+{\varvec{e}} \end{aligned}$$

where ${\varvec{b}}$ is the vector of random incomplete block effect following var(${\varvec{b}}$)$\sim$N(0,G5), where G5 is the variance-covariance structure for incomplete block effects following

$$\begin{aligned} G5=\begin{bmatrix} \sigma _{b_1}^2 &{}\ldots &{}\sigma _{b_1 b_n} \\ \vdots &{}\ddots &{}\vdots \\ \sigma _{b_n b_1} &{}\ldots &{}\sigma _{b_n}^2\\ \end{bmatrix}\otimes {\varvec{I}} \end{aligned}$$

where $\sigma _{b_1}^2$ and $\sigma _{b_n}^2$ are incomplete block variances for the 1st and the *n*th trait, and $\sigma _{b_1 b_n}$ and $\sigma _{b_n b_1}$ are incomplete block covariances between the 1st and the *n*th trait.

Trait narrow-sense heritability was estimated as follows:

$$\begin{aligned} h^2=\frac{\sigma ^2_a}{\sigma ^2_a+\sigma ^2_e} \end{aligned}$$

The pair-wise marker-based genetic correlation was estimated in terms of Pearson’s product moment as follows:

$$\begin{aligned} r_g=\frac{\sigma _{a_xa_y}}{\sqrt{\sigma ^2_{a_x}\sigma ^2_{a_y}}} \end{aligned}$$

where $\sigma _{a_x a_y }$ is the additive genetic covariance between trait x and y explained by genetic markers, and $\sigma _{a_x}^2$ and $\sigma _{a_y}^2$ are the additive genetic variances for trait x and y explained by markers. The standard errors of all genetic parameters were estimated through ’delta method’ based on Taylor expansion^50^. The similarity of correlation matrices between samples was compared using the Krzanowski test^51^ measuring the similarity between first principal components by using ’KrzCor’ function implemented in ’evolQG’ R package^52^ as follows:

$$\begin{aligned} r_{Krz}=\frac{1}{k} \sum _{i=1}^k\sum _{j=1}^k cos^2 (\Lambda _i^A,\Lambda _j^B) \end{aligned}$$

where k is the number of principal components derived from eigendecomposition of the additive genetic variance-covariance matrix and considered in test $k=\frac{n}{2}-1$ where n is the number of traits used in the multivariate analysis, and $\Lambda _i^A$ is the *i*th principal component of the additive genetic variance-covariance matrix of the *A*th sample. The phenotypes in the multivariate analysis used to construct additive genetic variance-covariance matrix were standardized in this case.

Selection response decomposition^53^ was performed to investigate similarity/dissimilarity between pairs of variance/covariance matrices obtained for each tested population and identify traits which cause differences in these matrices. The method implements the decomposition of evolutionary responses inferred from selection gradient vectors simulated through random skewers^54^. The similarity in response to selection between two investigated genetic variance/covariance matrices is estimated as the average correlation between vectors of evolutionary responses inferred from the same simulated vectors of selection gradients. The multivariate response to selection is estimated as follows:

$$\begin{aligned} \begin{bmatrix} \Delta _{z_1} \\ \Delta _{z_2} \\ \vdots \\ \Delta _{z_n} \end{bmatrix} = \begin{bmatrix} G_{11}\beta _1+G_{12}\beta _2+\ldots +G_{1n}\beta _n\\ G_{21}\beta _1+G_{22}\beta _2+\ldots +G_{2n}\beta _n\\ \vdots \\ G_{n1}\beta _1+G_{n2}\beta _2+\ldots +G_{nn}\beta _n\\ \end{bmatrix} \end{aligned}$$

where ${\varvec{\Delta _z}}$ is the vector of response to selection, ${\varvec{G}}$ is the additive genetic variance/covariance matrix, ${\varvec{\beta }}$ is the simulated vector of directional selection gradients. The identification of statistically different traits between the investigated populations is then performed through the decomposition of the above-mentioned product of the variance/covariance matrix ${\varvec{G}}$ and the vector of directional selection gradients ${\varvec{\beta }}$ to trait specific vectors of response as follows:

$$\begin{aligned} \begin{bmatrix} G_{11}\beta _1+G_{12}\beta _2+\ldots +G_{1n}\beta _n\\ G_{21}\beta _1+G_{22}\beta _2+\ldots +G_{2n}\beta _n\\ \vdots \\ G_{n1}\beta _1+G_{n2}\beta _2+\ldots +G_{nn}\beta _n\\ \end{bmatrix} \Rightarrow \begin{matrix} (G_{11}\beta _1+G_{12}\beta _2+\ldots +G_{1n}\beta _n)\\ (G_{21}\beta _1+G_{22}\beta _2+\ldots +G_{2n}\beta _n)\\ \vdots \\ (G_{n1}\beta _1+G_{n2}\beta _2+\ldots +G_{nn}\beta _n)\\ \end{matrix} \end{aligned}$$

The correlations of trait-specific vectors are estimated across tested populations for all simulated scenarios, and their average is called the SRD score. If ${\varvec{G}}$ matrices are different, the traits will show a different response to direct or indirect selection and result in low SRD score and large variance in correlations. The opposite trends will be observed in case when ${\varvec{G}}$ matrices are similar.

The prediction of correlation breakers was performed through logistic regression, where the aim was to predict the binary status of the correlation breakers through the genetic markers. The individuals originating from the correlation breakers (POP1HD) population were marked as 1 while individuals from POP1GF population were marked as 0. The POP2GF population was then used as an independent population to identify correlation breakers through the genomic prediction model. The logistic regression was performed in the ‘ASReml-R’ statistical package^47^ as follows:

$$\begin{aligned} {\varvec{y}}={\varvec{X\beta }}+{\varvec{Zu}}+{\varvec{e}} \end{aligned}$$

where ${\varvec{y}}$ is the vector of binary responses defining correlation breaker status, ${\varvec{\beta }}$ is the vector of fixed effects, and ${\varvec{u}}$ is the vector of pedigree or marker-based breeding values following var(${\varvec{u}}$)$\sim$N(0,${\varvec{A}}\sigma _u^2$), where ${\varvec{A}}$ is the average numerator relationship matrix which is substituted by the marker-based relationship matrix ${\varvec{G}}$ when genomic estimated breeding values are predicted. The leave-one-out strategy was selected to perform an independent evaluation due to the restricted number of individuals. The prediction accuracy was estimated as follows:

$$\begin{aligned} r=cor({\varvec{EBV}},{\varvec{GEBV}}) \end{aligned}$$

where ${\varvec{EBV}}$ is a vector of pedigree-based estimated breeding values, and ${\varvec{GEBV}}$ is a vector of predicted genomic breeding values. In the case of correlation breaker status (binary trait), two scenarios of cross-validation were performed: leave-one-out at (1) individual level (the same strategy as described above for quantitative traits); (2) family level where each time a whole single family was replaced as missing data to be predicted. The prediction accuracy was estimated in terms of area under the ROC (Receiver operating characteristic) curve (AUC). The AUC is the measure of the ability of a classifier to distinguish between classes (correlation breaker versus common individual status) and is used as a summary of the ROC curve. The higher the AUC, the better the performance of the model at distinguishing between the statuses of correlation breaker or common individual.

**Figure 1:**
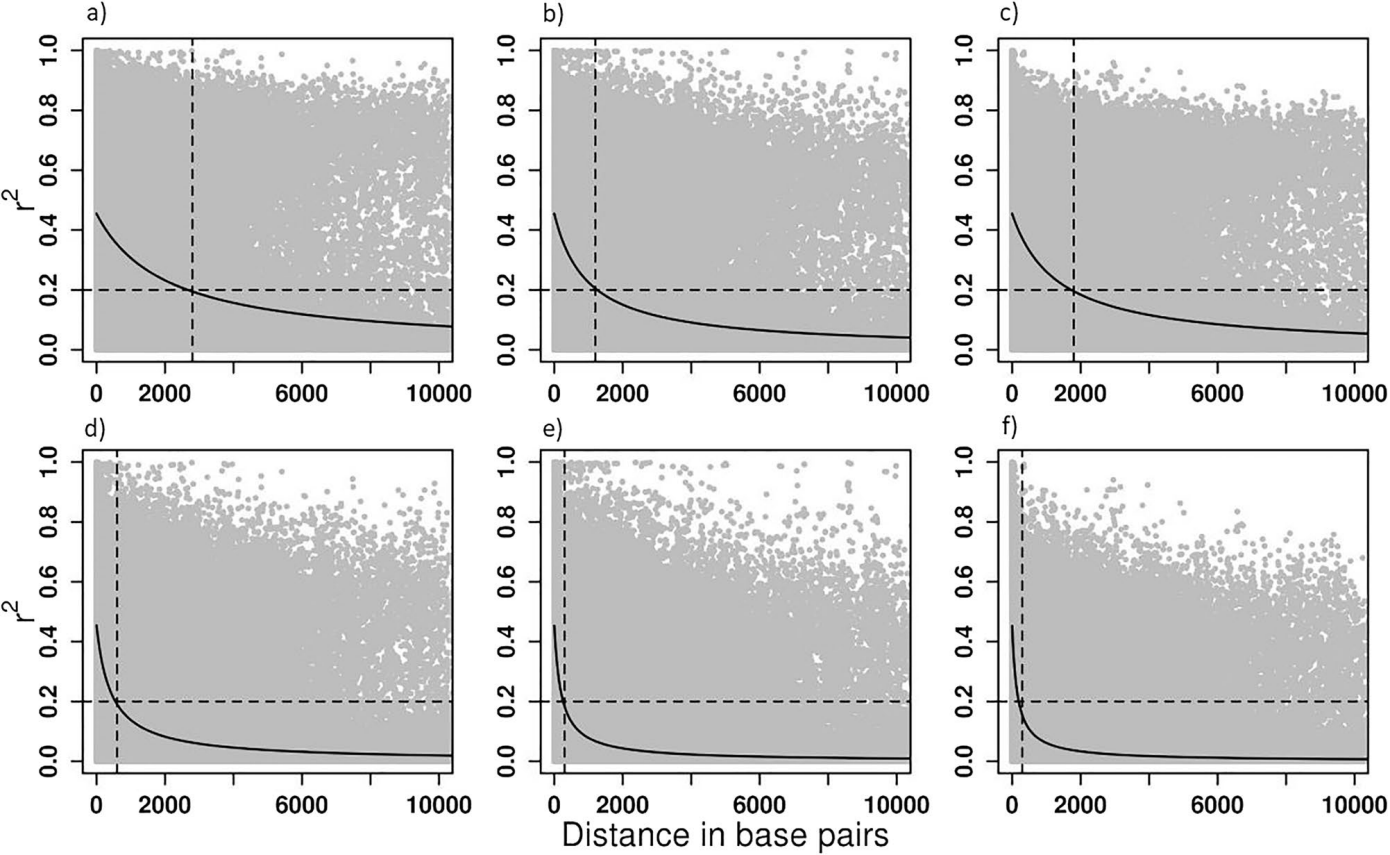
Linkage disequilibrium decay in: (**a**) POP1GF population; (**b**) POP1HD population; (**c**) POP2GF population and linkage disequilibrium decay corrected for bias due to familial relatedness in: (**d**) POP1GF population; (**e**) POP1HD population; (**f**) POP2GF population.

**Figure 2:**
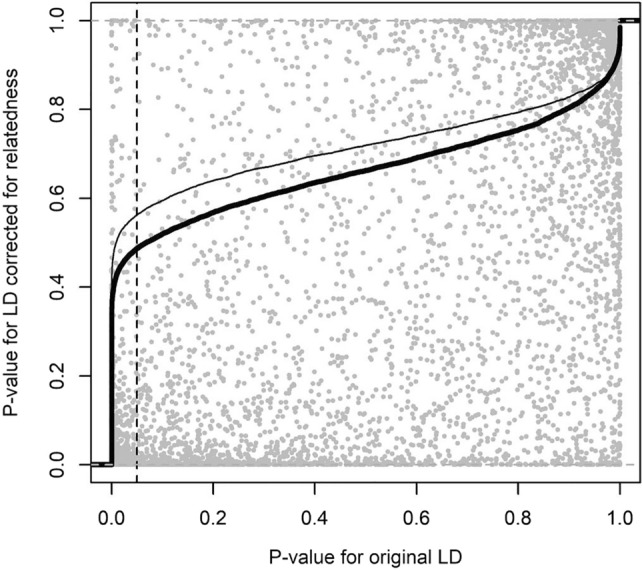
Correspondence in p-values from Jennrich test (test for difference in LD pattern between POP1GF and POP1HD) using original LD (including familial relatedness effect) and LD corrected for bias caused by familial relatedness. The thick line represents cumulative distribution of p-values for differences in LD pattern between scaffolds from POP1GF and POP1HD using unbiased LD estimate while thin line is cumulative distribution of *p* values for the same test using original LD (including effect of familial relatedness) Dashed line represents the threshold for statistically significant test.

**Figure 3:**
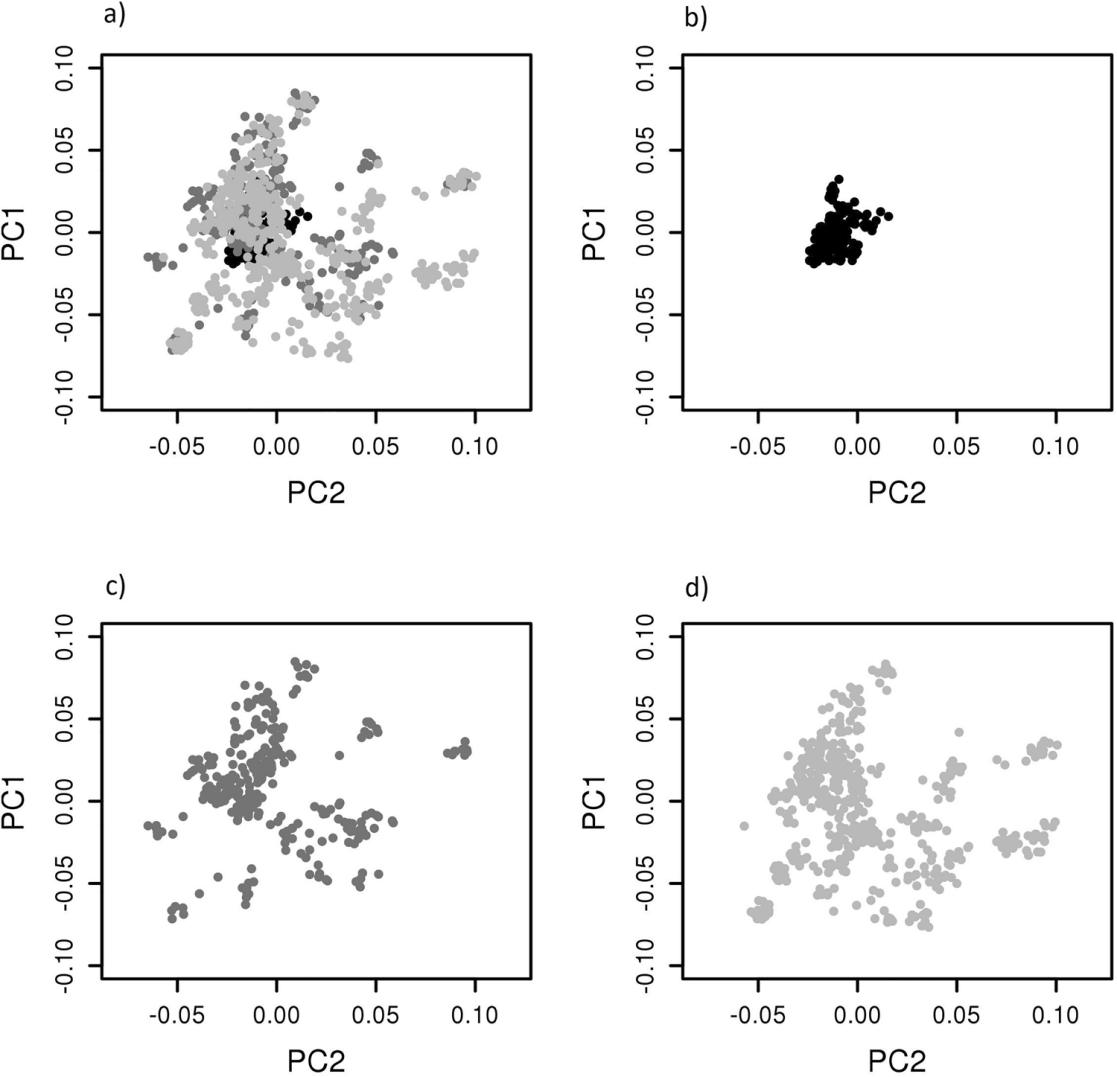
Population structure represented by the first and second components of the marker-based relationship matrix spectral decomposition: (**a**) across all population; (**b**) POP1HD population; (**c**) POP1GF population and (**d**) POP2GF population.

**Figure 4:**
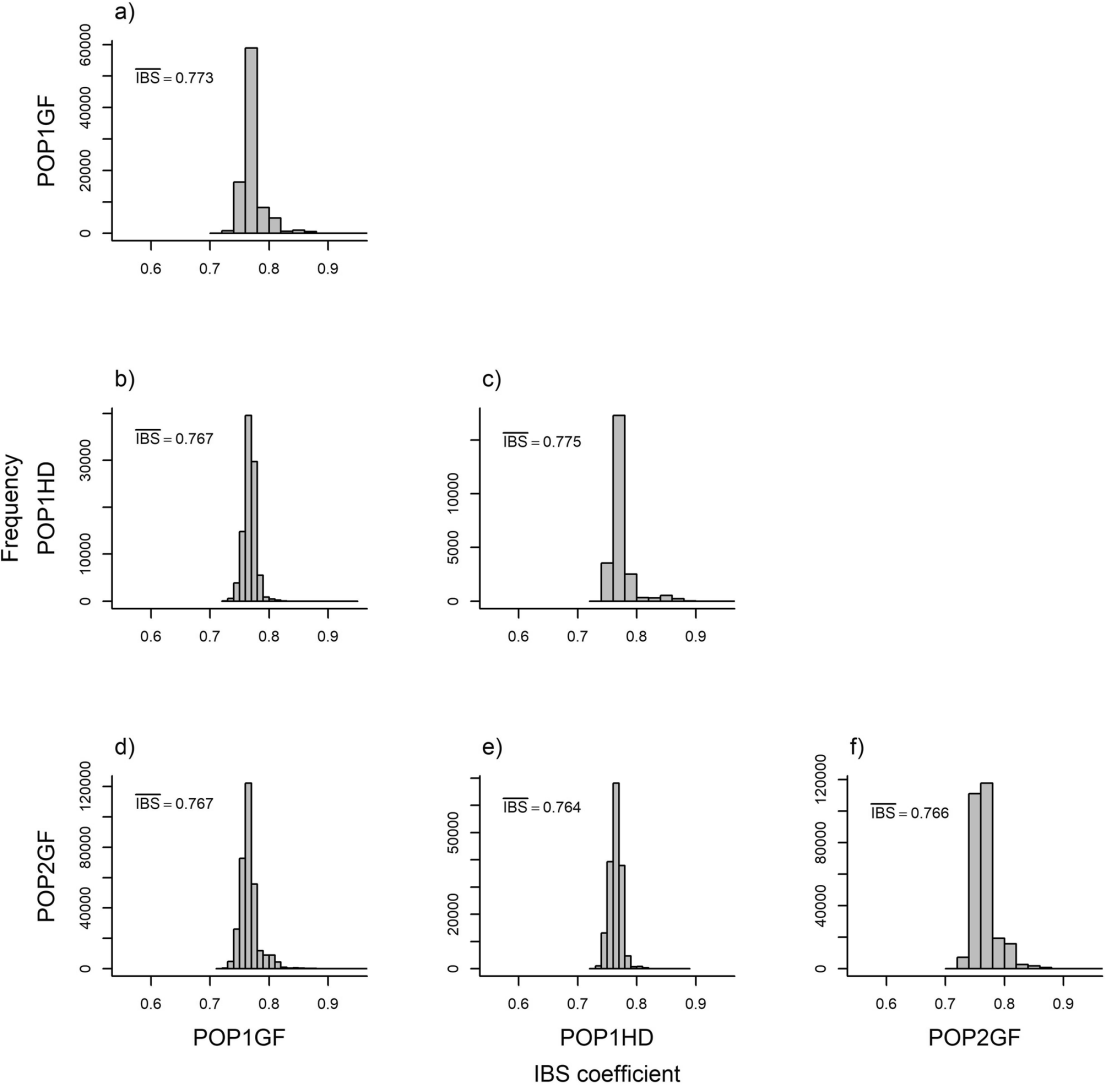
Distribution of identity by state (IBS) coefficients: (**a**) within POP1GF population; (**b**) between POP1GF and POP1HD population; (**c**) within POP1HD population; (**d**) between POP1GF and POP2GF population; (**e**) between POP1HD and POP2GF population and (**f**) within POP2GF population.

**Table 1:** Pedigree-based genetic correlation estimates and their standard errors (in parentheses).

Site	Population	BR9-DBH	BR9-ST9	BR9-WD	DBH-ST9	DBH-WD	ST9-WD
Tarawera	POP1GF	0.356 (0.115)	0.635 (0.131)	0.121 (0.131)	0.109 (0.132)	− 0.388 (0.103)	0.039 (0.132)
Tarawera	POP1HD	0.141 (0.139)	0.829 (0.087)	0.154 (0.166)	0.130 (0.168)	0.026 (0.136)	0.220 (0.143)
Woodhill	POP2GF	0.075 (0.128)	0.188 (0.150)	− 0.216 (0.159)	0.188 (0.150)	− 0.466 (0.175)	0.332 (0.186)
Kinleith	POP2GF	0.460 (0.146)	0.354 (0.163)	0.101 (0.092)	0.134 (0.221)	− 0.343 (0.130)	− 0.145 (0.130)

### Ethics approval and consent to participate

The study complies with Scion internal rules and guidelines for field operations and sampling of genetic material. All permissions required for data collection and sampling of plant tissues for DNA extraction were obtained. There is no permission required for the research on radiata pine in New Zealand.

## Results

### Linkage disequilibrium

Linkage disequilibrium (LD) decay was investigated within scaffolds which contained at least three Single Nucleotide Polymorphisms (SNPs), and that mapped to the loblolly pine (*Pinus taeda*) reference genome v. 1.01e. We found intensive LD decay in the correlation breaker population (POP1HD) where a composite estimate of linkage disequilibrium $r^2$ of 0.2 (considered as the threshold beyond which LD has been completely eroded^55^) was reached at 1 kb. By comparison, this same level of $r^2$ was reached at $\sim$2.5 kb in the POP1GF population and $\sim$ 2.1 kb in the POP2GF population. Therefore, the population of correlation breakers appeared to capture a higher amount of recombination events compared with the other two samples (Fig. 1). When LD was corrected for bias produced by familial relatedness, the observed linkage disequilibrium followed a similar patterns as before (i.e., including bias) but showed even faster decay along the scaffolds: the threshold of 0.2 was reached within $\sim$ 0.3 kb in correlation breakers (POP1HD) and within $\sim$ 0.6 kb in POP1GF population, while POP2GF reached $r^2$ of 0.2 within $\sim$ 0.3 kb (Fig. 1). Investigation of differences in LD patterns between the populations included in the training set (i.e., POP1GF and POP1HD) found that around 57% (LD including familial structure) and 48% (LD corrected for familial relatedness) of scaffolds had statistically significant differences in LD patterns. Therefore, familial relatedness contributed about 9% of statistically significant differences in LD patterns between populations. The correspondence in statistical significance between LD estimated (biased versus unbiased by familial relatedness) was relatively stable at the most significant and the most non-significant case with large changes in the middle of the distribution (Fig. 2). Spectral decomposition of the marker-based relationship matrix estimated across all samples showed that the POP1HD population created a compact cluster of related individuals when compared with populations selected for only growth and form. These correlation breakers are positioned at the centre of the investigated space (Fig. 3).

The statistical significance of the difference in effective population sizes between POP1GF and POP1HD was investigated using the resulting vectors of observed and/or expected heterozygosity in a *t* test. This analysis found statistically significant differences in both the mean observed as well as the expected heterozygosity between POP1GF and POP1HD. Although the mean expected heterozygosity was similar between both populations (POP1HD versus POP1GF) and reached values from 0.236 to 0.243, the average observed heterozygosity was lower in POP1HD (0.203) compared with POP1GF (0.226). Additionally, we investigated observed heterozygosity in markers from genomic regions with distinct LD pattern between POP1HD and POP1GF (based on results from the Jennrich test) and found higher observed heterozygosity of 0.23 compared to whole-genome observed heterozygosity of 0.203 in POP1HD compared to 0.22 which is similar to the whole-genome observed heterozygosity of 0.226 in POP1GF. Moreover, identity by state (IBS) coefficients were estimated to look at genetic similarity, unbiased by allelic frequencies. We found only subtle differences between the distributions of IBS within each investigated population as well as between them. While the average IBS coefficient within populations ranged from 0.766 (POP2GF) to 0.775 (POP1HD), slightly lower average IBS coefficients were found between populations ranging from 0.764 (POP1HD–POP2GF) to 0.767 (POP1GF–POP1HD and POP1GF–POP2GF) (Fig. 4).

### Genetic parameters

Both pedigree and marker-based analyses were able to recover statistically significant variance components and heritability estimates across all traits and populations/sites. The lowest heritability was observed for straightness (ST9), estimated to be 0.093 (POP2GF in Woodhill) − 0.212 (POP1HD) in the pedigree-based analysis. The highest heritability was observed for wood density (WD), reaching 0.350 (POP2GF in Kinleith) − 0.585 (POP1HD) when estimated from the pedigree across all samples. The marker-based analysis showed a similar pattern with the lowest heritability estimated for ST9, ranging from 0.087 (POP2GF in Kinleith) − 0.225 (POP1GF), and highest heritability estimated for WD, ranging from 0.518 (POP1GF) − 0.608 (POP2GF in Kinleith) (Table S1).

**Figure 5:**
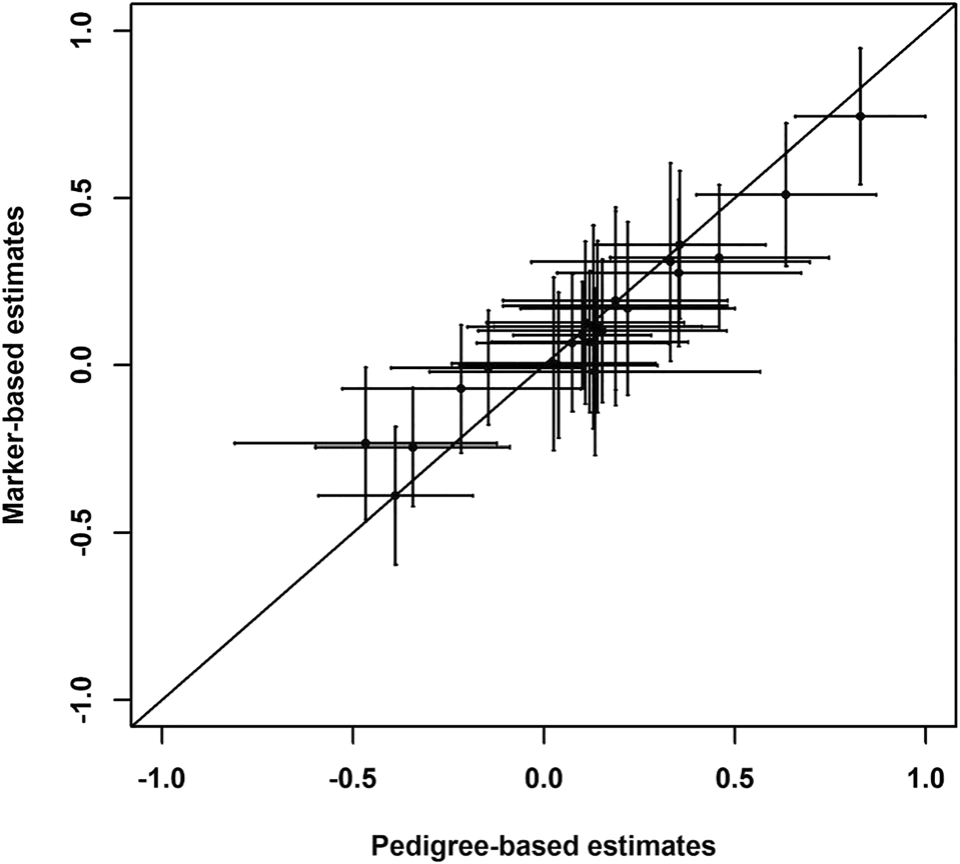
Correspondence pedigree-based and marker-based genetic correlations estimates.

Genetic correlations were estimated within each population at each site separately using both pedigree and marker-based analyses. These correlations serve as input parameters to compare differences in the multivariate response to selection between populations and environments. The genetic correlations generally corresponded across the methods. However, the 95% confidence limits were larger in pedigree-based estimates compared to marker-based equivalents, especially in the middle part of the distribution (Tables 1, 2; Fig. 5). The weakest genetic correlations were found between BR9 and DBH in POP2GF in Kinleith (0.061) and between DBH and WD in POP1HD (0.026), while the strongest genetic correlations were found between BR9 and ST9 in POP1HD (0.829) and in POP1GF (0.635) in pedigree-based analysis. It is worth noting that moderate negative correlations were observed between DBH and WD in all populations except for the above mentioned POP1HD, which reached values from − 0.318 to − 0.441 (Table 1). When marker-based analysis was implemented, the weakest genetic correlations were found between ST9 and WD in POP1GF (− 0.001) and between DBH and WD in POP1HD (0.004), while the strongest genetic correlations were found between BR9 and ST9 in POP1HD (0.774) and POP1GF (0.509) (Table 2). Substantial differences were observed in the estimation of genetic correlations at the sample level. The Krzanowski test (Table 3) found large differences between correlation matrices obtained in the correlation breaker population (POP1HD) compared with both other populations (POP1GF and POP2GF) across all sites. The correlation between correlation matrices obtained in POP1HD and POP1GF reached only 0.090, compared to 0.107 and 0.315 obtained between POP1HD and POP2GF. As expected, the highest correlations have been achieved between POP1GF and POP2GF, reaching from 0.770 to 0.913, since these two populations have undergone a similar selection history (Table 3).

**Figure 6:**
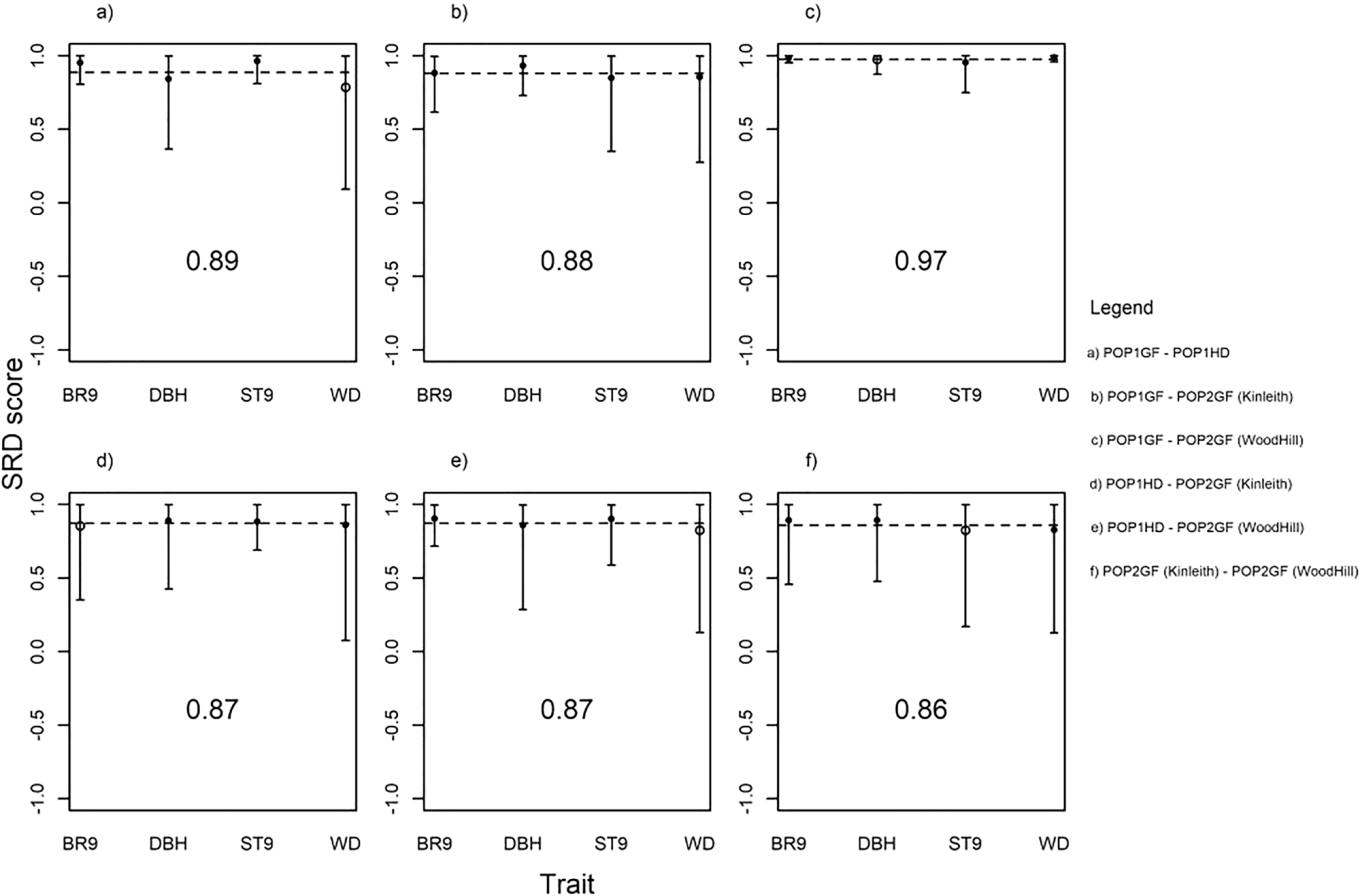
Selection response decomposition (SRD) score for the combination of each population sample under study: (**a**) POP1GF and POP1HD; (**b**) POP1GF and POP2GF at Kinleith; (**c**) POP1GF and POP2GF at Woodhill; (**d**) POP1HD and POP2GF at Kinleith; (**e**) POP1HD and POP2GF at Woodhill and (**f**) POP2GF at Kinleith and POP2GF at Woodhill. Mean is shown as a dotted line.

Selection response decomposition^53^ based on simulated vectors of selection responses was generally in an agreement between genetic variance-covariance matrices and differences appeared to be caused by environmental rather than genetic heterogeneity. For example, POP1GF and POP2GF, which are both tested in Woodhill and share the highest number of parents, also showed the highest correspondence among the estimates. On the other hand, the lowest correspondence among estimates was obtained between POP2GF tested in two different environments, which indicates the high impact of GxE on the genetic correlation matrix. The lowest correspondence of estimates between the identical genetic material planted at two different environments indicates strong influence or environmental conditions, especially on traits related to growth and stem form. Surprisingly, at high correspondence among estimates was also found between POP1GF and POP1HD, populations with different selection histories but planted in the same environment. A low correspondence was estimated among the POP1HD and POP2GF populations compared with the correspondence among the POP1GF and POP2GF populations, which could be attributable to the difference in selection history (Fig. 6).

**Table 2:** Marker-based genetic correlation estimates and their standard errors (in parentheses).

Site	Population	BR9-DBH	BR9-ST9	BR9-WD	DBH-ST9	DBH-WD	ST9-WD
Tarawera	POP1GF	0.360 (0.113)	0.509 (0.109)	0.069 (0.108)	0.127 (0.124)	− 0.390 (0.105)	− 0.001 (0.111)
Tarawera	POP1HD	0.115 (0.131)	0.744 (0.104)	0.102 (0.109)	0.114 (0.155)	0.004 (0.132)	0.169 (0.132)
Woodhill	POP2GF	0.066 (0.105)	0.193 (0.136)	− 0.071 (0.097)	0.176 (0.151)	− 0.234 (0.116)	0.308 (0.151)
Kinleith	POP2GF	0.321 (0.111)	0.275 (0.112)	0.089 (0.082)	− 0.020 (0.127)	− 0.245 (0.090)	− 0.008 (0.087)

### Genomic prediction accuracy

**Figure 7:**
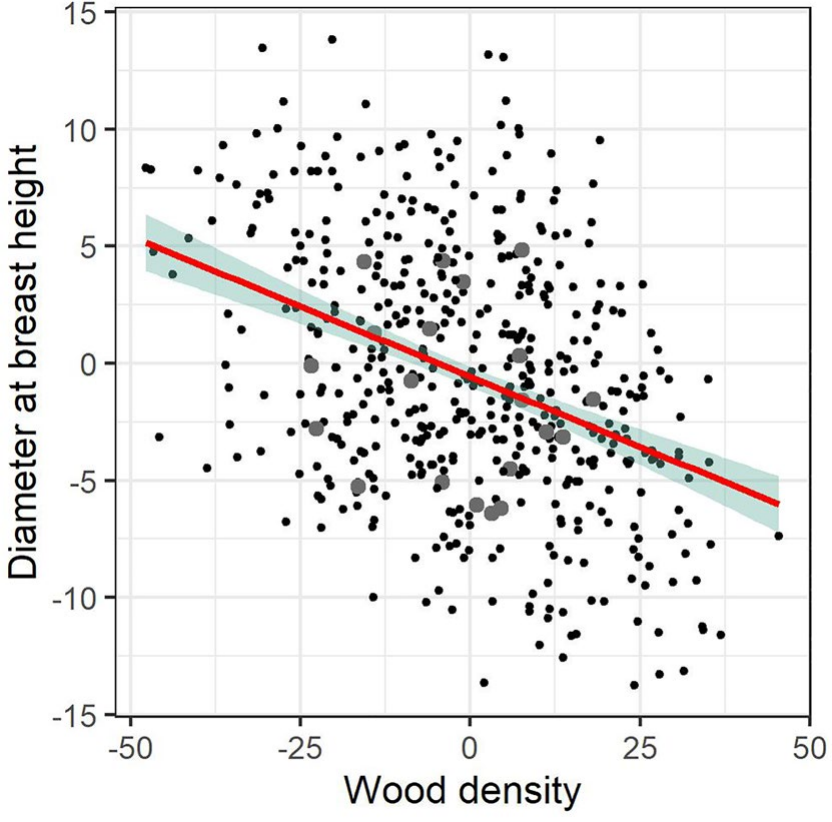
Distribution of individuals regarding their breeding values for DBH and WD; grey points indicate individuals predicted as correlation breakers in population POP2GF.

A genomic prediction model using correlation breaker status as a binary trait was implemented in two scenarios: (1) testing prediction accuracy of correlation breaker status within a training population that combined POP1HD and POP1GF, and (2) identifying correlation breakers in POP2GF. The ability to predict the status of correlation breakers in term of prediction accuracy was high and reached 0.80 when leave-one-out scenario was implemented and 0.72 when whole family was removed the from training population and their status predicted. Analysis of the POP2GF population resulted in the identification of 19 individuals with a predicted GEBV higher than 0.75, based on their genomic profile and after back transformation. When these individuals are plotted against their breeding values for DBH and WD, the predicted correlation breakers are in the centre of the distribution, with some cases that are superior for both traits and some that are inferior for both traits (Fig. 7). This might be caused by GxE interaction. The identification of correlation breaker status was performed on the GEBV identified at the 95 percentile of cumulative distribution of genomic breeding values. Since there was 35% of correlation breakers in training population, the 95 percentile represents very conservative approach to detect individuals with excess of heterozygosity within the genomic regions involving pleiotropic or collocating QTLs for investigated traits. In our case, the threshold GEBV value corresponding to 95 percentile was 0.75.

**Table 3:** Krzanowski correlations of marker-based (below diagonal) and pedigree-based (above diagonal) additive genetic variance-covariance matrices between samples.

Site		Tarawera	Tarawera	Woodhill	Kinleith
	Sample	POP1GF	POP1HD	POP2GF	POP2GF
Tarawera	POP1GF	0	0.090	0.913	0.770
Tarawera	POP1HD	0.024	0	0.107	0.315
Woodhill	POP2GF	0.816	0.179	0	0.795
Kinleith	POP2GF	0.823	0.227	0.968	0

## Discussion

### Evolutionary advantages of correlation breakers

Our study investigated the potential of genomic selection to identify individuals with high levels of recombination once the proper training population is established. These individuals are called “correlation breakers” and usually represent individuals that deviate strongly from general trends in genetic correlation between traits (traits that usually have unfavourable genetic relationships). We proposed that the identification of such individuals will lead to improvements in the response to selection in both traits, when traits are adversely correlated, due to decayed genetic correlation. We also postulated that high recombination might also improve population resilience to climate change. Under random mating, the cause of genetic correlations is proposed to be the result of mainly pleiotropic mutations^4^. In populations under non-random mating, genetic correlations are the results of LD between loci affecting the correlated traits, causing inbreeding^6^. However, the process of adaptation to local environmental conditions may result in the development of genomic rearrangements, such as inversions^56^, that produce clusters of tightly linked adaptive loci and are referred as super-genes^57^. Such genomic rearrangements can potentially reshape the network of genetic correlations between adaptive traits. Genetic correlations represent evolutionary constraints, and their unfavourable combination can compromise progress in evolutionary changes. This can be detrimental under intensive environmental changes that require a fast genetic response. Theoretical studies^58^ have shown the evolutionary advantages of recombination due to the ability of linked loci to interfere with each other’s response to selection.

Additionally, an excess of recombination events has been shown to be favourable under heterogeneous environmental conditions^14^. Correlation breakers represent individuals that have accumulated an excess of recombination events, and propose their infusion into breeding program/forest plantations to support population resilience under changing environmental conditions. This is especially important in forest trees, as they are long-living organisms facing temporal variability in environmental conditions along their ontogenetical development, exacerbated by climate change. Mathematical modelling found an advantage of recombination at the early stage of adaptation, while selection against recombination was pronounced at later stages to eliminate maladaptive gene flow^12,59^. Additionally, the empirical study in lodgepole pine did not find any higher recombination rates between genes associated to different aspects of current climate patterns^60^. However, if we assume continuously changing environmental conditions with a higher frequency of extreme weather and climate events^61,62^, evolutionary processes might change direction which would shift back to the early stage of adaptation, and an excess of recombination would be advantageous. The decomposition of contemporary genetic correlations will not only release new genetic variation but also increase progress in evolutionary responses to new environmental conditions. Nevertheless, such a putative positive effect is restricted only to the genetic correlations caused by LD, while those caused by pleiotropic mutations should remain unaffected by recombination rates. However, pleiotropy-related genetic correlations could still be influenced by new environmental conditions through other mechanisms^9^.

The identification of individuals with an excess of recombination events is not straightforward since LD decay is a population-specific parameter which depends on breeding and selection history^63^. Therefore, the establishment of a specific population containing correlation breakers is required. Such populations can be created through tandem selection as implemented in this study, or by complementary mating where the best performing individuals for each unfavourably correlated trait are mated^15^. Therefore, being a correlation breaker is more a relative concept than heritable feature. However, our analysis of observed heterogeneity discovered that, while the population of correlation breakers showed lower levels of heterozygosity across the whole genome, possibly due to directional selection for both traits (DBH and WD), they also showed increased level of observed heterozygosity in regions with statistically different patterns in LD. These regions might contain groups of pleiotropic or collocating QTLs for selected traits. Our multivariate analysis discovered that the tandem selection strategy disrupted most of the commonly expected correlations in the population apart from the correlation among BR9 and ST9 (Tables 1, 2). Such results are probably the product of the pleiotropic architecture of the traits rather than a function of the genetic correlations themselves^64^. The clear distinction in networks of genetic correlation was confirmed in the results from the Krzanowski test^51^. However, a more obvious pattern was observed in the marker-based compared with pedigree-based analysis. This shows the strong ability of markers generated through exome capture genotyping by sequencing to track differences in selection and breeding history compared with the expected genealogy captured by pedigrees. While the information included in pedigrees is limited by the definition of the base population (pedigree founders) as having with no relatedness and inbreeding, the markers can trace both the relatedness created recently within breeding program and also relatedness and population structure that existed prior to the formation of the base population^65^. Additionally, genomic markers enable tracing of the Mendelian sampling term and linkage disequilibrium between QTLs and markers as oppose to the expected identity by descent derived from pedigree information^66^. On the other hand, such a clear pattern was not observed in the selection response decomposition, where the differences between correlation patterns appeared to be caused by environmental rather than genetic heterogeneity (Fig. 5). However, the sample tested in field experiments had passed several cycles of selection, and thus the genetic diversity needed for robust estimates of genetic correlation was limited, in addition to the contribution of the small sample size to the unclear pattern in genetic correlation comparisons^67^.

The establishment of a robust training population is crucial to be able to predict individuals with a high levels of recombination events (correlation breakers). Our training population was established from sets of progenies derived from parents selected with a tandem selection strategy, and progeny of parents from a directional selection strategy. The ability to distinguish LD decay pattern developed from exome capture markers (Fig. 1) was crucial to clearly identify the individuals belonging to the correlations breakers set when population structure was investigated (Fig. 2). The accuracy of predictions for correlation breaker status was high, reaching 0.80, which provides strong evidence to support the selection of individuals with an excess of recombination events. Such individuals are preferred under heterogeneous environmental conditions^14^, and their infusion into populations should help erode unfavourable genetic correlations and initiate faster evolutionary processes, in response to climate change. The genomic selection prediction model was also able to identify 19 individuals as having correlation breaker status in an independent population (POP2GF). These individuals are centred in the middle of the distribution with regard to breeding values for DBH and WD, with some having breeding values above average in both traits to some having breeding values below average in both traits (Fig. 6). However, since the LD pattern is population specific, the status of correlation breakers has to be considered in the context of the training population and corresponds to the level of genetic diversity present. Therefore, genetically broader samples should be used in training and in the definition of correlation breakers.

### Mitigation of climate change through genomics

Forest trees are mostly widespread species occupying geographically large and environmentally highly heterogeneous areas which, accompanied with intensive long-distance gene flow, should support the rapid adaptation of populations to new environmental conditions^68^. However, there are concerns that such natural mechanisms are not fast enough to cope with the current speed of climate change without human intervention, and a more active approach needs to be applied. An assisted gene flow approach^69^ was proposed as a mechanism to shift current populations towards their future optimal conditions as predicted by climate models^70–73^. The current development of genomic resources in forest trees enables a deeper insight into adaptation processes through the detection of selection signals at the genome level^23,74,75^. However, adaptive traits are rather complex, and capturing selection signals through association mapping can be challenging^76^. Therefore, genomic selection^77^ or genome-wide selection scans^78,79^ based on the deployment of genetic markers to predict phenotype through multivariate regression models seems to be a more feasible solution to predict adaptive traits. Arenas et al.^80^ proposed genomic prediction of putative adaptive traits in small natural population of relict species to optimize conservation management.

Genomic selection has been successfully implemented in animals^81,82^, agriculture crops^83,84^ and forest trees^85–91^. Compared to animals and plants, however, forest trees have high genetic diversity, extreme genome lengths (e.g., $\sim$ 25 Gb in radiata pine), rapid LD decay^23,92^, and breeding programs are generally only in their early stages due to late expression of sexual maturity and long breeding cycles. All these factors pose challenges to the implementation of genomic prediction in forest trees. Grattapaglia and Resende^93^ performed a deterministic simulation of scenarios relevant to forest trees and found that a sufficient density of markers is a crucial requirement to perform genomic prediction successfully.

The extreme length of the genome of forest trees prohibits easily capturing the whole genome through whole genome re-sequencing, and genotyping platforms based on reduced representation approaches have to be deployed^36,94^. Since prediction models can get overwhelmed with the exhaustive amount of genomic data^66^, the effort invested into the reduction of genome complexity should not be seen not as a handicap, but rather as a challenge to identify relevant genomic fragments. Our study is based on genomic resources that were developed from the sequencing of transcriptomes from a range of different tissues, including buds, needles, xylem, and phloem^38^. These genomic resources generated $\sim$ 800 K SNPs representing $\sim$ 40 K genes. However, most SNPs were rare variants, likely due to the fact that the exome is a highly conserved region, and only $\sim$ 80 K SNPs were used to generate genotypes for each individual.

The radiata pine genomic resources generated within our project were assembled against the loblolly pine draft reference genome^95^, which allowed us to look at LD decay with physical distance. The population of correlation breakers (POP1HD) showed much more rapid LD decay compared with the other samples (POP1GF and POP2GF; Fig. 1). However, all samples appeared to aggregate in a cloud around $r^2$ of 0.6 in the correlation breakers (POP1HD) and POP2GF, and around 0.7 in POP1GF across the investigated range of physical distances (Fig. 1). This is likely due to differences in synteny between the loblolly pine and radiata pine genomes. The estimates of 1 kb, 2.1 kb and 2.5 kb around an $r^2$ of 0.2 in the correlation breakers and the other two populations (Fig. 1) are probably upwardly biased due to low sample sizes^96^ and the actual decay in linkage disequilibrium is most likely more intensive (especially in the correlation breakers population where the sample size was even lower). Chao et al.^63^ found differences in the long-range level of LD between different wheat populations and attributed it to differences in breeding and selection history. However, they did not find any differences in LD decay, and each population captured a comparable amount of recombinant events.

In contrast, our samples do show a difference in LD decay, with the fastest decay apparent in the correlation breakers population, which provides evidence that a higher amount of recombination events is being captured in this sample compared with others. This pattern is expected, as the correlation breakers population was selected to break the adverse pattern in genetic correlations between two highly complex traits, which would require a higher level of recombination between a large number of QTLs associated with the different traits. Additionally, the increased recombination rate in this population could stimulate an increased response to selection and a decreased loss of additive genetic variance over time^82^.

Since the expectation for $r^2$ is $E(r^2 )=\frac{1}{1+4Nc}$ where N is effective population size and c is recombination rate, the difference in the effective population size investigated through the mean observed heterozygosity was statistically significant among populations and thus N is likely to contribute to the LD estimate. This was especially observed when LD was corrected for bias due to familial relatedness, resulting in much faster decrease in LD decay in POP1GF compared to POP1HD. However, even after correcting for LD bias due to familial relatedness, POP1HD showed LD that decayed twice as fast as POP1GF. Additionally, correction of LD for familial relatedness resulted in only 9% decrease in the number of scaffolds showing statistically significant difference in LD patterns between POP1GF and POP1HD (Fig. 2), and thus difference in recombination rates is likely the driver of differences in LD patterns. The lower mean observed heterozygosity in correlation breakers population can be seen as contradictory to the notion that the higher level of heterozygosity is favourable in heterogeneous environmental conditions^97^. The reduced level of heterozygosity can be connected to accumulation of favourable alleles for QTLs involved in genetic architecture of both traits under selection (DBH and WD). Additionally, despite the relatively high conservedness of exome regions, as evidenced by similar IBS within and between populations—Fig. 7, we were able to track differences in both general LD decay patterns and differences in local LD patterns between investigated populations to identify individuals with high levels of recombination (Figs. 1, 2).

The key advantage of the genomics-based approach in breeding is the recovery of both temporal and historical relatedness in studied populations^65^ through the construction of a marker-based relationship matrix^41,98^. The implementation of this type of relationship matrix in genetic evaluations involves the simple substitution of the pedigree-based alternative and does not require any additional data treatment. The spectral decomposition of the marker-based relationship matrix found a strong difference in population structure between the correlation breakers that were selected for HD and other samples selected for GF. While samples selected for GF show high dispersion across the investigated space, the correlation breakers population was concentrated in the middle (Fig. 2). Therefore, it is likely that stronger selection in the trait with a higher heritability (HD) is reflected in the reduced genetic diversity of POP1HD sample. As reported in this study, genetic correlation networks change with changes in environmental conditions. This might be the consequence of gene x environment interactions and the resulting impact on multivariate responses. Although the QTL effects might fluctuate with changes in environmental conditions, we assume the underlying genetic architecture will remain the same. Thus, the selection for increased heterozygosity in genomic regions accumulating pleiotropic or collocating QTLs for traits under selection might be an effective way to decompose unfavourable genetic correlations. The tandem selection implemented in the radiata pine breeding program proved to be a successful approach to decompose unfavourable genetic correlations and stimulate multivariate response to selection. However, we expect that the efficiency of this approach will depend on the genetic architecture involved in genetic correlations. In cases, where pleiotropic effects are the primary driver of genetic correlations, the tandem selection might be less efficient compared to cases where genetic correlation is caused by collocating QTLs.

## Conclusions

We propose that environment-specific (representing the average New Zealand environment) selection be undertaken using genomic predictions by increasing the frequency of ’recombined’ individuals. We propose that high levels of recombination will also confer long-term resilience in planted forests, vital to the success of these populations under a changing climate. The increased frequency of such individuals may decrease the strength of the population-level genetic correlations among traits, which will, in turn, increase the opportunity for new trait combinations to be developed in the future.

## Supplementary Information

Supplementary Information.
